# Inflammasome Signaling in Cardiac Arrhythmias: Linking Inflammation, Fibrosis, and Electrical Remodeling

**DOI:** 10.3390/ijms26135954

**Published:** 2025-06-20

**Authors:** Paschalis Karakasis, Konstantinos Pamporis, Panagiotis Theofilis, Nikias Milaras, Panayotis K. Vlachakis, Konstantinos Grigoriou, Dimitrios Patoulias, Theodoros Karamitsos, Antonios P. Antoniadis, Nikolaos Fragakis

**Affiliations:** 1 Second Department of Cardiology, Hippokration General Hospital, Aristotle University of Thessaloniki, 54124 Thessaloniki, Greece; aantoniadis@gmail.com (A.P.A.); fragakis.nikos@googlemail.com (N.F.); 2 First Cardiology Department, School of Medicine, Hippokration General Hospital, National and Kapodistrian University of Athens, 15772 Athens, Greece; konstantinospab@gmail.com (K.P.); panos.theofilis@hotmail.com (P.T.); nikiasmilaras@gmail.com (N.M.); vlachakispanag@gmail.com (P.K.V.); 3 Department of Pharmacology, University of Athens, 11527 Goudi, Greece; dinosgrigoriou@gmail.com; 4 Second Propedeutic Department of Internal Medicine, Faculty of Medicine, School of Health Sciences Aristotle, University of Thessaloniki, 54642 Thessaloniki, Greece; dipatoulias@gmail.com; 5 First Department of Cardiology, Aristotle University Medical School, AHEPA University General Hospital, 54636 Thessaloniki, Greece; tkaramitsos@auth.gr

**Keywords:** atrial fibrillation, ventricular arrhythmias, NLRP3 inflammasome, cardiac remodeling, calcium handling, inflammation, fibrosis, SGLT2 inhibitors

## Abstract

Cardiac arrhythmias, including atrial fibrillation and ventricular arrhythmias, remain leading causes of morbidity and mortality worldwide. While structural, electrical, and metabolic remodeling have long been recognized as drivers of arrhythmogenesis, emerging evidence identifies inflammation—particularly inflammasome signaling—as a central orchestrator of this pathological triad. Among the various inflammasome complexes, the NLRP3 inflammasome has garnered particular attention due to its activation in cardiomyocytes, fibroblasts, and immune cells in diverse clinical contexts. NLRP3 activation precipitates a cascade of downstream events, including interleukin-1β and -18 maturation, oxidative stress amplification, calcium mishandling, and extracellular matrix remodeling, thereby fostering a proarrhythmic substrate. This review synthesizes mechanistic and translational data implicating inflammasome signaling in both atrial and ventricular arrhythmias, with a focus on cellular specificity and electrophysiological sequelae. We explore upstream triggers, such as metabolic stress, gut dysbiosis, and epicardial adipose inflammation, and delineate the downstream impact on cardiac conduction and structural integrity. Emerging therapeutic strategies—including NLRP3 inhibitors, IL-1 antagonists, colchicine, and SGLT2 inhibitors—are critically appraised for their anti-inflammatory and antifibrotic potential. By bridging molecular insights with clinical application, this review underscores the inflammasome as a unifying mechanistic hub in arrhythmia pathogenesis and a promising target for precision-guided therapy.

## 1. Introduction

Cardiac arrhythmias, particularly atrial fibrillation (AF) and ventricular arrhythmias, represent a major global health burden, contributing substantially to morbidity, mortality, and healthcare utilization [1,2,3,4,5,6,7,8,9]. Although structural, electrical, and metabolic remodeling have long been recognized as key contributors to arrhythmogenesis, a growing body of evidence now places inflammation—specifically inflammasome signaling—at the center of this pathophysiological landscape [10]. Among the various inflammasomes identified to date, the nucleotide-binding domain, leucine-rich repeat-containing receptor pyrin domain containing three (NLRP3) inflammasomes has gained particular attention due to its involvement in numerous cardiovascular conditions, including AF, heart failure, and post-infarction remodeling [10,11,12,13,14,15,16].

While traditionally associated with immune cells, emerging evidence suggests that inflammasome activation also occurs in nonimmune cell populations, including cardiomyocytes and fibroblasts, underscoring its broad biological relevance in cardiac disease [17]. Within the arrhythmogenic substrate, NLRP3 activation has been implicated in a range of pathological processes—including calcium-handling abnormalities, oxidative stress, cytokine production, gap junction remodeling, and fibrosis—each of which contributes to the initiation and maintenance of abnormal cardiac rhythms [18,19,20]. Notably, the inflammasome operates not as a solitary effector but as a nodal integrator of diverse stress signals, thereby serving as a mechanistic bridge linking metabolic, electrophysiological, and structural perturbations [21,22,23].

This review aims to critically examine the mechanistic role of NLRP3 inflammasome activation in the pathogenesis of atrial and ventricular arrhythmias. We synthesize current evidence from experimental and clinical studies, outline cell-specific signaling mechanisms, and evaluate emerging therapeutic strategies targeting inflammasome pathways. By integrating insights from molecular immunology and cardiac electrophysiology, we highlight the translational potential of inflammasome modulation in arrhythmia prevention and management.

## 2. Inflammasome Overview: Classification and Mechanisms of Activation

Inflammasomes are cytosolic multiprotein complexes that function as critical components of the innate immune response, acting as pattern recognition platforms for the detection of pathogenic microorganisms and endogenous danger signals [24,25]. These structures orchestrate the inflammatory cascade by sensing pathogen-associated molecular patterns (PAMPs)—such as lipoteichoic acid, lipopolysaccharides, and peptidoglycans—as well as damage-associated molecular patterns (DAMPs), thereby initiating a robust host defense mechanism [26]. While primarily associated with immune cells, inflammasome activation has also been observed in various non-immune cellular populations, reflecting its broad functional relevance in tissue homeostasis and injury response [16,24,27,28].

To date, at least ten distinct inflammasome sensors have been identified, including interferon-γ-inducible protein 16 (IFI16), absent in melanoma 2 (AIM2), and several members of the nucleotide-binding domain, leucine-rich repeat-containing (NLR) family such as NLRP1, NLRP2, NLRP3, NLRP6, NLRP12, and NLRC4 [26,29,30]. Among these, the NLRP3 inflammasome has emerged as the most extensively characterized and clinically significant due to its involvement in a diverse array of inflammatory and cardiovascular pathologies, including atrial fibrillation [31].

Activation of the NLRP3 inflammasome is a tightly regulated two-step process comprising a priming phase and an activation (triggering) phase [25,32]. During priming, the engagement of toll-like receptors (TLRs) by DAMPs or PAMPs stimulates the NF-κB signaling pathway, leading to the transcriptional upregulation of NLRP3 itself, as well as the inactive pro-forms of interleukin (IL)-1β and IL-18 [33,34]. The subsequent activation phase involves assembly of the inflammasome complex through interactions between NLRP3, the adaptor protein ASC (apoptosis-associated speck-like protein containing a caspase recruitment domain), and pro-caspase-1 [33,35]. This oligomerization event culminates in the autocatalytic cleavage of caspase-1, which in turn processes pro-IL-1β and pro-IL-18 into their mature, biologically active forms—key mediators of systemic and localized inflammation [33,35]. This canonical activation cascade constitutes the molecular foundation through which inflammasomes execute their immunologic and pathogenic functions [33].

Calcium (Ca^2+^) is a fundamental second messenger involved in a wide array of biochemical and physiological processes, including signal transduction, gene expression, and immune regulation [36]. Emerging evidence suggests that intracellular Ca^2+^ mobilization plays a pivotal role in the activation and assembly of the NLRP3 inflammasome [37]. Both extracellular influx and the intracellular release of Ca^2+^ contribute to the elevation of cytosolic Ca^2+^ concentrations, thereby facilitating inflammasome activation [37].

One of the key mechanisms underlying this process involves the calcium-sensing receptor (CaSR), predominantly expressed on the surface of immune cells. Upon engagement by extracellular Ca^2+^, CaSR activates downstream signaling cascades, notably phospholipase C (PLC), which catalyzes the production of inositol 1,4,5-trisphosphate (IP_3_) [38]. IP_3_, in turn, binds to its receptors (IP_3_Rs) located on the sarcoplasmic/endoplasmic reticulum, triggering the release of stored Ca^2+^ into the cytoplasm [39]. Concurrently, receptor-operated Ca^2+^ entry channels facilitate further Ca^2+^ influx from the extracellular space [39]. This concerted elevation in intracellular Ca^2+^ appears to be a critical permissive event for the oligomerization and activation of the NLRP3 inflammasome complex [39]. Notably, the pharmacologic inhibition of IP_3_R-mediated Ca^2+^ release using agents such as 2-aminoethoxydiphenyl borate (2-APB) significantly attenuates cytosolic Ca^2+^ levels, concomitantly suppressing the maturation and secretion of IL-1β [39].

Elevated cytosolic Ca^2+^ concentrations are closely linked to secondary increases in mitochondrial Ca^2+^ uptake, a process that precipitates the generation of mitochondrial reactive oxygen species (ROS) [40,41]. This surge in ROS serves as a critical signal for the activation of the NLRP3 inflammasome, highlighting a mechanistic interplay between Ca^2+^ homeostasis, mitochondrial dysfunction, and innate immune activation [40,41]. Accordingly, oxidative stress emerges as a potent upstream instigator of inflammasome assembly and function [40,41]. Systemic oxidative stress has been identified as a robust biomarker of AF risk, reflecting upstream cardiometabolic burden and serving as a key amplifier of redox-sensitive pathways such as NLRP3 inflammasome activation [42]. Its association with atrial remodeling and arrhythmia susceptibility further supports its relevance in the inflammatory substrate of AF [42]. In parallel, the Ca^2+^-responsive phosphatase calcineurin is activated in the setting of increased intracellular Ca^2+^, further amplifying the inflammatory response [40,41]. Experimental models have demonstrated that the cardiac-specific overexpression of PPP3CA, the gene encoding the catalytic subunit of calcineurin, leads to the pronounced upregulation of NLRP3 expression, enhanced cleavage and activation of CASP1, and elevated circulating levels of IL-1β [43].

## 3. Inflammatory Mechanisms Underlying Atrial Fibrillation Pathogenesis

Inflammation represents a fundamental defense mechanism activated in response to tissue injury or infection [44]. However, when persistent, it becomes maladaptive, driving pathological remodeling within the atrial substrate [44]. Chronic inflammation promotes the sustained production of ROS and proinflammatory cytokines, which in turn facilitate atrial fibrosis, cellular hypertrophy, and apoptosis—hallmark processes in the initiation and maintenance of atrial cardiomyopathy (AtCM) and AF [45,46,47,48]. The disruption of laminar blood flow within the atria contributes to endothelial microinjury, facilitating the transmigration of immune cells into atrial tissue [49,50,51]. Evidence from histopathologic analyses of atrial samples obtained from 46 patients undergoing valvular or coronary artery bypass surgery revealed the significantly increased infiltration of CD68-KP1^+^ inflammatory cells—predominantly dendritic cells—and CD3^+^ T-lymphocytes in the left atrial myocardium of patients with AF compared to those in sinus rhythm [52]. Notably, this immune cell infiltration was independently associated with arrhythmic status, irrespective of comorbid conditions such as hypertension, DM, systemic inflammatory markers, or anatomical variations including age and atrial size [52]. These findings point to a rhythm-specific inflammatory response, with the preferential accumulation of monocyte–macrophage lineage cells [52]. This localized immune activation fosters cytokine release, structural remodeling, and subsequent arrhythmogenic substrate formation [52]. Importantly, macrophage infiltration appears to precede, rather than result from, chemokine signaling, suggesting an initiating role in the inflammatory cascade of AF [52,53,54]. Moreover, the histologic evaluation of atrial tissues from AF patients consistently reveals low-grade inflammation characterized by leukocyte and macrophage infiltration [49,50,51].

Accumulating evidence implicates inflammation—particularly inflammasome activation within atrial cardiomyocytes—as a critical driver of AF development and progression [55,56]. The inflammasome is present in both immune and nonimmune cell types, which orchestrates the innate immune response to infectious and sterile stimuli [57]. It comprises three essential components: a pattern recognition receptor (typically a nucleotide-binding oligomerization domain-like receptor, NLR), the adaptor protein ASC, and an effector cysteine protease, most commonly CASP1 or CASP5 [57].

Among the various inflammasome subtypes, NLRP3, NLRP1, and NLRC4 are the most well-characterized, each defined by the specific NLR protein that confers signal specificity [58,59]. In atrial cardiomyocytes, inflammasome activation results in the cleavage and activation of CASP1, which in turn processes the pro-forms of IL-1β and IL-18 into their mature, bioactive counterparts [58,59]. The subsequent increase in circulating IL-1β and IL-18 levels correlates with the transition from paroxysmal to persistent or chronic AF and is mechanistically linked to AF and structural remodeling [58,59]. The pathogenic interplay among cardiomyocytes, fibroblasts, and immune cells establishes a self-perpetuating proinflammatory and profibrotic circuit [58,59]. Immune cell infiltration into atrial tissue leads to the release of inflammatory cytokines that inflict injury upon cardiomyocytes [58,59]. In response, damaged cardiomyocytes activate intracellular innate immune pathways, including NLRP inflammasome signaling, thereby amplifying the local inflammatory milieu through further IL-1β and IL-18 secretion [58,59]. Simultaneously, the inflammatory environment and cardiomyocyte death promote fibroblast activation, facilitating extracellular matrix deposition and fibrotic remodeling [58,59]. These activated fibroblasts, in turn, exacerbate immune cell recruitment via chemokine and cytokine secretion, sustaining and propagating atrial inflammation and fibrosis [58,59,60,61].

Beyond its metabolic functions, epicardial adipose tissue (EAT) has emerged as an active paracrine and immunomodulatory organ implicated in the pathophysiology of AF [62]. EAT secretes a spectrum of proinflammatory adipocytokines, including IL-6, IL-8, IL-1β, and TNF-α, which diffuse into the adjacent atrial myocardium and create a localized proinflammatory microenvironment [63,64,65]. This cytokine milieu facilitates the recruitment and activation of immune cells within the atrial tissue, thereby promoting fibroblast activation and contributing to the fibrotic remodeling of the atrial substrate. Moreover, epicardial adipocytes exhibit anatomic and functional plasticity, with the capacity to infiltrate the underlying myocardium [66,67,68]. This ectopic adipose infiltration disrupts the structural integrity and electrophysiological properties of the atrial myocardium, leading to conduction heterogeneities that predispose to AF initiation and maintenance [64].

EAT, anatomically contiguous with the myocardium and devoid of fascial separation, exerts direct paracrine influence on adjacent cardiac structures [69]. Alterations in EAT volume and morphological characteristics have been closely associated with a spectrum of metabolic and cardiovascular disorders [69]. In a clinical investigation involving 152 patients—categorized into diabetic, obese, and lean cohorts and undergoing valve repair and/or coronary artery bypass grafting—paired blood and subcutaneous adipose tissue (SAT) samples were obtained [69]. Analysis revealed that EAT from diabetic and obese individuals exhibited a marked upregulation of proinflammatory cytokines, including IFN-γ, TNF-α, IL-6, and IL-1, highlighting a state of heightened inflammatory activation within this fat depot [69]. The study further demonstrated that both EAT and SAT harbor adaptive immune cell populations, suggesting a broader immunologic functionality beyond passive fat storage [69]. Notably, the inflammatory signaling profile of EAT was significantly altered in the context of diabetes, obesity, and associated cardiometabolic perturbations, reinforcing its role as a dynamic immunometabolic organ. To functionally assess the impact of EAT-derived inflammation on atrial remodeling, SAT and EAT specimens from 39 patients undergoing coronary bypass surgery were used to establish a rat atrial organoculture model [70]. This model revealed that the secretome of EAT—rich in bioactive molecules such as activin A, a profibrotic adipokine—exerted potent fibrogenic effects on atrial myocardium [70]. These findings implicate EAT-derived paracrine factors in promoting myocardial fibrosis, thereby contributing to the structural remodeling that underlies AF susceptibility.

The expansion of EAT, frequently observed in the context of obesity, has been increasingly recognized as a contributor to the proinflammatory milieu that fosters AF development. To elucidate the electrophysiological consequences of EAT on atrial cardiomyocytes, an experimental model utilizing rabbit left atrial myocytes was employed [71]. Cardiomyocytes were harvested and subsequently co-cultured with adipocytes derived from EAT [71]. Compared to control cardiomyocytes, those exposed to epicardial adipocytes exhibited significant alterations in membrane electrophysiology, including a more depolarized resting membrane potential and augmented L-type Ca^2+^ and Na^+^ current densities [71]. These changes are indicative of heightened excitability and altered ionic homeostasis—key substrates for arrhythmogenesis [71]. These findings suggest that adipocytes resident in EAT exert direct modulatory effects on atrial electrophysiology, thereby promoting an arrhythmogenic phenotype [72]. Through both paracrine signaling and potential cellular interactions, EAT may play a mechanistically active role in AF pathophysiology by destabilizing atrial electrical properties and facilitating the initiation and maintenance of the arrhythmia [72,73,74].

### 3.1. NLRP3 Inflammasome Activation as a Driver of Electrophysiological Remodeling in Atrial Fibrillation

**Figure 1:**
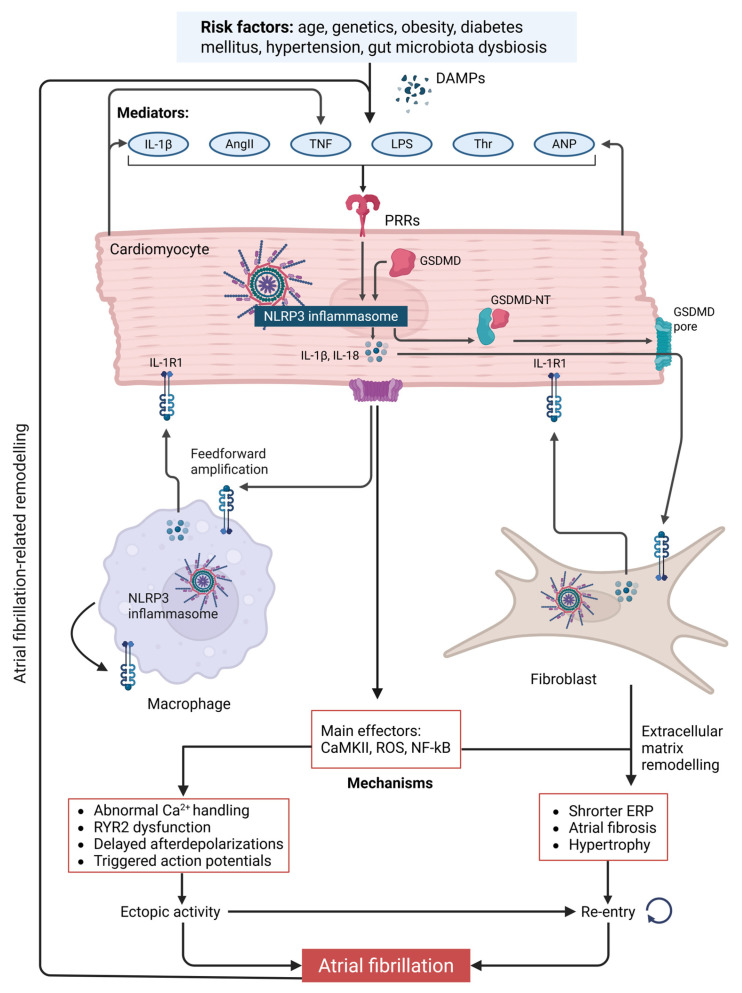
Role of cardiomyocyte–immune cell crosstalk in inflammasome-mediated atrial fibrillation pathophysiology. In the context of atrial fibrillation (AF), dynamic interactions between cardiomyocytes and immune cells contribute to the establishment and perpetuation of a proinflammatory arrhythmogenic substrate. AF-promoting stressors activate pattern recognition receptors (PRRs) on cardiomyocytes, leading to assembly of the NLRP3 inflammasome and subsequent maturation of interleukin (IL)-1β and IL-18. These cytokines, along with the downstream activation of Ca^2+^/calmodulin-dependent protein kinase II (CaMKII), reactive oxygen species (ROS), and nuclear factor-κB (NF-κB) signaling, promote pathological remodeling. IL-1β further activates IL-1 receptor type 1 (IL-1R1) on adjacent macrophages, amplifying inflammatory signaling, while IL-1R1 stimulation on fibroblasts drives extracellular matrix remodeling. This inflammatory milieu results in Ca^2+^ handling abnormalities within cardiomyocytes, including ryanodine receptor 2 (RyR2) dysfunction, delayed afterdepolarizations, and triggered action potentials. These changes, coupled with effective refractory period (ERP) shortening and structural fibrosis, promote the initiation and maintenance of AF. Abbreviations: AngII, angiotensin II; ANP, atrial natriuretic peptide; DAMPs, damage-associated molecular patterns; GSDMD, gasdermin D; GSDMD-NT, gasdermin D N-terminal fragment; LPS, lipopolysaccharide; NLRP3, NACHT, LRR and PYD-containing protein 3; Thr, thrombin; and TNF, tumor necrosis factor.

Among the various inflammasome complexes, the NLRP3 inflammasome is the most extensively characterized and has been implicated in the pathogenesis of a wide spectrum of cardiovascular disorders, including AF [75] (Figure 1). The constitutive or aberrant activation of NLRP3 has been documented in patients with paroxysmal, chronic, and postoperative AF, suggesting a conserved proarrhythmic role across clinical AF phenotypes [76]. A gain-of-function mutation in NLRP3 (A350V), as modeled in NLRP3^A350V/+^ knock-in mice, leads to heightened assembly and activation of the inflammasome complex [77]. In a seminal study by Yao et al. [19], atrial tissue samples and isolated cardiomyocytes from patients with paroxysmal or chronic AF revealed elevated NLRP3 inflammasome activity. To further delineate causal mechanisms, a cardiomyocyte-specific knock-in mouse model expressing NLRP3^A350V/+^ under the control of the α-myosin heavy chain promoter was developed [19]. These mice exhibited spontaneous premature atrial contractions, inducible AF, and a marked increase in NLRP3-mediated inflammatory signaling within atrial cardiomyocytes [19]. Crucially, the pharmacologic inhibition of NLRP3 with MCC950 suppressed the arrhythmic phenotype, underscoring the functional relevance of inflammasome activity in AF [19]. In this model, NLRP3 activation was linked to profound disturbances in intracellular Ca^2+^ homeostasis, most notably aberrant sarcoplasmic reticulum (SR) Ca^2+^ dynamics driven by the upregulated expression of ryanodine receptor 2 (RyR2) [19]. In parallel, electrical remodeling was characterized by action potential duration (APD) shortening—a substrate conducive to reentrant arrhythmias—associated with the increased expression of atrial-selective ultra-rapid delayed-rectifier (I_Kur) and acetylcholine-activated inward-rectifier (I_K,ACh) potassium currents [19]. These electrophysiological changes were accompanied by structural remodeling, including atrial hypertrophy and a marked reduction in the effective refractory period (ERP), further facilitating arrhythmogenesis [19]. These perturbations collectively created an atrial substrate conducive to arrhythmia initiation and maintenance [19]. Electrophysiologic studies in the knock-in model further confirmed increased Ca^2+^ spark frequency, disrupted electric activation patterns, and reduced the atrial ERP, all of which were concordant with findings in human AF tissue samples [19]. These data suggest that aberrant SR Ca^2+^ dynamics, driven by NLRP3 inflammasome activation, are central to the proarrhythmic remodeling of atrial tissue [19].

In a transgenic mouse model of spontaneous atrial fibrillation driven by cardiac-specific overexpression of the cAMP response element modulator (CREM), targeted silencing of Nlrp3 using the adeno-associated virus serotype 9 (AAV9)-mediated delivery of small interfering RNA (siRNA) effectively abrogated the development of AF [19]. These findings provide compelling in vivo evidence that NLRP3 inflammasome activation plays a causative role in AF pathogenesis. Importantly, this study positions NLRP3 inhibition as a potentially viable therapeutic strategy for the prevention or attenuation of AF, particularly in settings characterized by heightened inflammasome activity [19]. Nevertheless, further mechanistic investigations are warranted to delineate the precise molecular crosstalk between cardiomyocyte NLRP3 signaling and sarcoplasmic reticulum Ca^2+^ handling, which may constitute a key axis in the arrhythmogenic remodeling underlying AF.

Postoperative AF remains a prevalent complication following cardiac surgery, yet the underlying molecular mechanisms are incompletely understood [78,79]. Emerging evidence now implicates aberrant inflammasome signaling in this context. In a recent investigation, atrial tissue homogenates and isolated cardiomyocytes from patients who developed postoperative AF exhibited a marked upregulation of NLRP3 inflammasome components, suggesting heightened innate immune activation in the atrial substrate [80]. To mechanistically model the postoperative inflammatory milieu, the acute administration of IL-1β—an effector cytokine of inflammasome activation—was applied to murine HL-1 atrial cardiomyocytes [80]. This intervention triggered robust NLRP3 inflammasome activation and led to the calcium/calmodulin-dependent protein kinase II (CaMKII)-mediated hyperphosphorylation of key calcium-handling proteins, including ryanodine receptor 2 (RYR2) and phospholamban (PLN) [80]. Consequently, sarcoplasmic reticulum (SR) Ca^2+^ leak was significantly enhanced in both HL-1 cells and atrial cardiomyocytes derived from postoperative AF patients. This aberrant Ca^2+^ release promoted delayed afterdepolarizations—proarrhythmic events known to predispose to AF initiation [80]. Collectively, these findings provide compelling evidence that exposure to proinflammatory cytokines such as IL-1β initiates a pathological cascade involving NLRP3 inflammasome activation and the CaMKII-dependent dysregulation of intracellular calcium homeostasis in atrial cardiomyocytes.

Moreover, while the NLRP3 inflammasome has been extensively implicated in AF, recent evidence highlights the pathogenic relevance of the absent in melanoma 2 (AIM2) inflammasome in AtCM and arrhythmogenesis. In a pivotal study, the dietary induction of a high-protein metabolic state was shown to promote an AF-prone atrial phenotype via activation of the AIM2 inflammasome [81]. This process was mechanistically linked to mitochondrial oxidative stress and pathological SR Ca^2+^ release events—both key drivers of atrial electrical instability [81]. These findings expand the current paradigm of inflammasome-mediated atrial remodeling by identifying AIM2 as a previously unrecognized contributor to AtCM and AF susceptibility. The parallel involvement of both NLRP3 and AIM2 inflammasomes in maladaptive atrial remodeling underscores their potential as upstream regulators of proarrhythmic signaling cascades and supports their candidacy as novel therapeutic targets in the prevention and management of AF.

Although cardiomyocytes have historically been regarded as the primary site of NLRP3 activation in arrhythmogenesis, recent data underscore the contributory roles of other myocardial cell types. Notably, fibroblast-restricted NLRP3 activation has been shown to promote atrial fibrillation and diastolic dysfunction via increased IL-1β secretion and intercellular crosstalk with cardiomyocytes, culminating in electrical remodeling and calcium mishandling [18]. Similarly, macrophage-mediated inflammation—particularly through IL-1β-driven mitochondrial ROS production and RyR2 destabilization—has been implicated in diabetes-associated AF [82]. These findings suggest that NLRP3 activation in non-cardiomyocyte populations can exert proarrhythmic effects, either directly or via the paracrine modulation of cardiomyocyte function. Thus, the arrhythmogenic potential of NLRP3 signaling appears to transcend cellular boundaries, reflecting an integrated network of immune and stromal interactions within the atrial substrate.

### 3.2. Proinflammatory Cytokine Signaling Downstream of Inflammasome Activation in Atrial Fibrillation

Beyond its canonical role in the maturation of IL-1β and IL-18, the NLRP3 inflammasome has been implicated in the upregulation of additional proinflammatory cytokines, including TNF-α and IL-6—both of which have been associated with AF pathophysiology [83,84,85,86]. Mounting evidence supports a mechanistic link between inflammasome-mediated cytokine production and atrial electrical and structural remodeling. In a comprehensive investigation by Yao et al. [87], heightened NLRP3 inflammasome activity was identified across multiple AF models, including murine models of spontaneous AF, canine models of atrial tachycardia, and atrial tissue from patients with paroxysmal and chronic AF [87]. Utilizing a cardiomyocyte-specific NLRP3 knock-in model, the study demonstrated that inflammasome activation led to the upregulation of CASP1, increased susceptibility to pacing-induced AF, and frequent premature atrial contractions [87]. Concomitantly, there was a marked elevation of IL-1β and COL1A1 mRNA expression, implicating inflammasome-driven macrophage activation and fibroblast-mediated fibrosis in the remodeling process [87]. Importantly, the genetic ablation of NLRP3 in this model reversed the arrhythmic phenotype and attenuated the associated proinflammatory and profibrotic responses, including IL-6 expression. These findings underscore the centrality of NLRP3-driven cytokine signaling—particularly IL-6—as a downstream effector in AF pathogenesis [87]. Therapeutic strategies aimed at inhibiting NLRP3 activation or neutralizing its effector cytokines represent a promising avenue for the mitigation of AF progression and recurrence.

The signal transducer and activator of transcription 3 (STAT3), a member of the STAT protein family, has emerged as a key transcriptional regulator implicated in the pathogenesis of AF [88]. The aberrant activation of STAT3 not only contributes directly to atrial structural remodeling but also promotes the transcriptional upregulation of NLRP3 via epigenetic modifications [89]. Specifically, STAT3 facilitates the acetylation of histones H3 and H4 at the NLRP3 promoter region, thereby enhancing its expression and downstream inflammasome activation [89]. In a rat model of sterile pericarditis, Huang et al. demonstrated the robust upregulation of IL1B, IL6, TGFB1, TNF, STAT3, and microRNA-21 (miR-21), suggesting widespread inflammatory and profibrotic activation in the atrial myocardium [90]. The pharmacologic inhibition of STAT3 using S3I-201 significantly suppressed miR-21 expression, mitigated atrial fibrosis, and reduced AF susceptibility. This intervention also downregulated canonical fibrotic genes including ACTA2, COL1A1, and COL3A1, and improved atrial conduction homogeneity [90]. Conversely, IL-6 stimulation of cultured cardiac fibroblasts led to increased STAT3 phosphorylation and upregulation of miR-21—an effect that was abrogated by STAT3 inhibition [90]. Moreover, the forced overexpression of miR-21 enhanced STAT3 activation and promoted the transcription of fibrotic genes, supporting the existence of a feed-forward loop between STAT3 and miR-21 that perpetuates fibrotic remodeling [90]. These findings delineate the IL-6–STAT3–miR-21 signaling axis as a pivotal pathway in AF pathophysiology. Given the established role of NLRP3 inflammasome activation in driving IL-6 production, it is plausible that inflammasome-mediated cytokine signaling acts upstream of STAT3-miR-21 activation. This integrated inflammatory-fibrotic circuit provides mechanistic insight into atrial substrate remodeling and presents multiple potential targets for therapeutic intervention in AF.

Tumor necrosis factor-alpha (TNF-α) is a pleiotropic proinflammatory cytokine that plays a prominent role in cardiovascular inflammation and arrhythmogenesis [91]. Beyond its classical immunomodulatory functions, TNF-α also regulates the transcriptional machinery responsible for the expression of inflammatory mediators and NLRP3 inflammasome components, thereby amplifying the innate immune response within the atrial myocardium [92].

Microarray-based transcriptomic analyses have implicated TNF-α in the pathogenesis of exercise-induced AF, revealing the activation of mitogen-activated protein kinase 14 (MAPK14) and nuclear factor-kappa B (NF-κB) signaling pathways [93]. In experimental models, the pharmacologic inhibition or genetic ablation of TNF (Tnf) or MAPK14 significantly attenuated AF onset and atrial remodeling following strenuous exercise, establishing a mechanistic link between TNF-α signaling and stress-induced arrhythmogenic remodeling [93].

Pulmonary vein (PV) cardiomyocytes—widely recognized as focal drivers of paroxysmal AF—appear particularly susceptible to TNF-α-induced electrophysiological derangements [94]. In a study by Lee et al. [95], the TNF-α stimulation of isolated rabbit PV cardiomyocytes resulted in pronounced delayed afterdepolarizations, elevated diastolic intracellular Ca^2+^ levels, and impaired SR Ca^2+^ handling [94]. Specifically, TNF-α exposure led to the downregulation of ATP2A2 (encoding SERCA2a), decreased SR Ca^2+^ content, and diminished transient Ca^2+^ amplitude—hallmarks of disrupted intracellular Ca^2+^ homeostasis and enhanced arrhythmogenic potential [94].

Complementary insights were provided by Sawaya et al. [96], who utilized a cardiac-specific TNF-α overexpression model in knock-in mice. Constitutive TNF-α activation led to marked conduction abnormalities in both atria and ventricles, accompanied by the downregulation of GJA5 (encoding connexin40), a key gap junction protein implicated in electrical coupling [96]. These structural and electrical alterations collectively contribute to a proarrhythmic substrate conducive to AF maintenance [96]. Taken together, these findings substantiate the role of TNF-α as a pathogenic effector in AF through its multifaceted actions on calcium handling, proinflammatory signaling, and electrical remodeling. The targeted inhibition of TNF-α, particularly within the PV cardiomyocyte population, represents a promising therapeutic strategy to mitigate AF susceptibility and progression.

### 3.3. NLRP3 Inflammasome Activation in Atrial Fibroblasts: Implications for Atrial Fibrosis

Fibroblasts, as resident sentinel cells within the cardiac interstitium, are highly responsive to their microenvironment and play a central role in the orchestration of fibrotic remodeling [97]. Accumulating evidence indicates that these cells contribute substantially to the development of the arrhythmogenic substrate in AF, particularly through their capacity to initiate and propagate fibrotic responses [98,99]. Upon activation, fibroblasts undergo phenotypic transformation into myofibroblasts—specialized cells with enhanced contractility and a pronounced capacity for extracellular matrix (ECM) deposition, including the synthesis of collagen [99].

Emerging studies have identified the NLRP3 inflammasome as a pivotal regulator of fibroblast function and fibrotic transformation [100]. Post-myocardial infarction models have shown significant upregulation of Nlrp3, Il1b, and Il18 mRNA in infarcted tissue compared with controls, with expression most prominent in non-cardiomyocyte cellular fractions [100]. In vitro, cardiac fibroblasts derived from sham-operated mice exhibited a robust induction of IL-1β and IL-18 following classical inflammasome priming and triggering via lipopolysaccharide and ATP stimulation, respectively [100]. Moreover, in human cardiac fibroblasts, the thrombin-mediated activation of NLRP3 signaling occurs through protease-activated receptor-4 (PAR4), further supporting the inflammasome’s functional relevance in fibroblast-mediated inflammation [76].

Interestingly, in murine models with cardiomyocyte-specific NLRP3 overexpression, the increased expression of fibrotic markers such as collagen-1α and galectin-3 suggests a paracrine mechanism by which cardiomyocyte inflammasome activation may secondarily stimulate NLRP3 signaling in adjacent fibroblasts [19]. Additionally, oxidative stress—a well-recognized driver of fibroblast proliferation and myofibroblast differentiation—has been implicated as a key upstream activator of NLRP3 in these cells [101]. Recent data suggest that fibroblast-restricted NLRP3 activation is sufficient to induce myofibroblast differentiation, enhance proliferative and contractile capacity, and promote fibrotic expansion, thereby contributing directly to atrial remodeling and AF pathogenesis [102].

Importantly, a landmark study by Li et al. [18] provided compelling in vivo evidence that fibroblast-specific NLRP3 inflammasome activation directly promotes atrial cardiomyopathy, arrhythmogenesis, and heart failure with preserved ejection fraction (HFpEF). Using a fibroblast-restricted knock-in (FB-KI) mouse model expressing constitutively active NLRP3, the investigators demonstrated that inflammasome activation enhanced fibroblast proliferation, myofibroblast transdifferentiation, collagen deposition, and impaired gap junction coupling—without eliciting systemic inflammation [18]. Optical mapping revealed impaired conduction velocity, and in vivo electrophysiology confirmed increased AF susceptibility [18]. Notably, AAV-mediated Nlrp3 silencing reversed atrial dysfunction, fibrosis, and AF incidence, establishing a causal link [18].

Together, these findings not only validate NLRP3 as a central mediator of fibroblast-driven fibrotic remodeling but also identify fibroblast-targeted inflammasome inhibition as a promising therapeutic strategy to counter atrial arrhythmogenesis and HFpEF progression. The distinction between cardiomyocyte- and fibroblast-specific inflammasome activation underscores the need for cell-type-specific modulation to effectively disrupt the multifaceted proarrhythmic cascade in AF. Table 1 presents key preclinical and translational studies that delineate the role of inflammasome activation in the pathogenesis of AF.

### 3.4. Gut Microbiota Dysbiosis and Inflammasome Activation in Atrial Fibrillation

The human gastrointestinal tract harbors a complex and dynamic ecosystem of microorganisms—including bacteria, archaea, and eukaryotes—collectively termed the gut microbiota [104,105]. This microbial community plays a vital role in maintaining host metabolic, immunological, and inflammatory homeostasis [104,105]. Perturbations in this ecosystem, referred to as gut microbiota dysbiosis, are characterized by reduced microbial diversity and an increased abundance of proinflammatory taxa and have been implicated in a range of systemic and cardiovascular diseases [106].

Recent evidence suggests a mechanistic link between gut dysbiosis and the pathogenesis of AF, potentially through inflammasome-mediated inflammatory pathways [107]. In a cohort study involving 50 individuals with AF, both serum and fecal analyses revealed marked alterations in microbial diversity and taxonomic composition, indicative of dysbiotic profiles [108]. Similarly, the comparative profiling of gut microbiota in 30 patients with paroxysmal AF, 20 with chronic AF, and 50 control subjects demonstrated that both AF phenotypes shared a common dysbiosis signature [108]. This altered microbial landscape is thought to contribute to systemic inflammation and may potentiate AF progression through modulation of the NLRP3 inflammasome and related inflammatory circuits [108].

In a pivotal study, Zhang et al. investigated the mechanistic role of gut microbiota dysbiosis in aging-associated AF utilizing a fecal microbiota transplantation (FMT) model in rats [109]. The transplantation of dysbiotic microbiota, particularly from aged donors, led to significant elevations in circulating glucose and lipopolysaccharide levels in recipient animals—biochemical alterations that coincided with the pronounced upregulation of NLRP3 inflammasome expression [109]. This inflammatory activation was mechanistically linked to the development of atrial fibrosis and increased AF susceptibility in the host. Pharmacological inhibition of the NLRP3 inflammasome using MCC950 attenuated both atrial fibrosis and arrhythmogenicity, underscoring the inflammasome’s functional role in mediating gut-derived inflammatory signaling in the atrial substrate [109]. Notably, these preclinical findings were corroborated by cross-sectional analyses in human AF cohorts, wherein gut dysbiosis correlated with systemic inflammation and atrial remodeling [110].

Collectively, these findings delineate a pathophysiological axis wherein gut microbiota dysbiosis contributes to aging-associated AF through glucose- and lipopolysaccharide-mediated activation of the NLRP3 inflammasome [27,55]. This gut-heart crosstalk highlights a dual-target therapeutic strategy—simultaneously restoring microbial homeostasis and inhibiting inflammasome activity—as a promising approach to mitigate AF in the elderly population.

### 3.5. Obesity-Induced Inflammation and Inflammasome Activation in Atrial Fibrillation

Obesity and AF represent two globally prevalent and interlinked epidemics, both of which substantially elevate the risk of cardiovascular morbidity and mortality [111]. Epidemiological evidence consistently demonstrates that individuals with excess body weight exhibit a markedly increased incidence, prevalence, and clinical severity of AF compared to their lean counterparts [112]. One of the central mechanistic links between obesity and AF lies in the transformation of adipose tissue phenotype and function [113]. In the context of visceral adiposity, metabolically active brown adipocytes are replaced by dysfunctional white adipocytes, which adopt a proinflammatory secretory profile [113]. These pathogenic adipocytes release a range of inflammatory cytokines that recruit immune cells into the adipose microenvironment, thereby establishing a chronic, low-grade inflammatory state [114].

This inflammation is not confined to adipose tissue but extends systemically, contributing to atrial structural and electrical remodeling—hallmarks of an arrhythmogenic substrate [114]. Localized inflammation within pericardial and epicardial adipose depots further exacerbates atrial fibrosis and conduction abnormalities. Increasingly, the NLRP3 inflammasome is recognized as a critical molecular mediator at the intersection of obesity-induced inflammation and AF pathophysiology [115,116]. Through its role in amplifying IL-1β and IL-18 signaling, NLRP3 activation links metabolic dysfunction to immune-driven atrial remodeling [115,116].

A compelling body of evidence has delineated a mechanistic link between obesity and AF, with a key role attributed to NLRP3 inflammasome activation within the atrial myocardium. In a translational study encompassing obese mice, sheep, and human subjects, obesity was consistently associated with heightened activation of the NLRP3 inflammasome across all three species, implicating a conserved inflammatory axis in obesity-induced atrial remodeling [103]. To specifically elucidate the role of NLRP3 in mediating obesity-related AF, NLRP3 knockout (NLRP3^−/−^) mice were subjected to either a standard chow or high-fat diet over a 10-week period [103]. In obese wild-type mice, atrial remodeling was characterized by the upregulation of KCNA5—encoding a potassium channel subunit critical for repolarization—resulting in ERP shortening [103]. Concomitantly, the dysregulated SR Ca^2+^ handling and activation of profibrotic signaling pathways were observed, collectively creating an arrhythmogenic substrate. Notably, these pathological alterations were mitigated in NLRP3^−/−^ mice, establishing a causal role for NLRP3 inflammasome activation in obesity-induced AF [103].

These findings position the atrial NLRP3 inflammasome as a pivotal mediator linking metabolic stress to electrical and structural remodeling of the atria [114]. As such, the targeted inhibition of NLRP3 may represent a promising therapeutic strategy to attenuate AF risk in the context of obesity. While considerable attention has been given to cardiomyocyte-specific NLRP3 activation in AF pathogenesis, the contribution of adipocyte-based inflammasome signaling—particularly within EAT—remains inadequately explored [117,118]. Given the close anatomic proximity and paracrine interaction between EAT and the underlying myocardium, further investigation into EAT-specific NLRP3 inflammasome activation is warranted [119]. Such studies may uncover novel inflammatory mechanisms by which EAT modulates atrial electrophysiology and fibrosis, offering new insights into obesity-related AF and opportunities for cell-type–directed therapeutic intervention.

### 3.6. Diabetes and Inflammasome Activation in Atrial Fibrillation

Diabetes mellitus (DM) is a well-established risk factor for AF, with epidemiological studies indicating that individuals with DM face approximately a 40% higher risk of developing AF compared to non-diabetic populations [120]. This elevated risk is compounded by the frequent co-occurrence of hypertension and vascular disease—conditions commonly linked to diabetic pathophysiology. A range of maladaptive processes, including electrical, structural, electromechanical, and autonomic remodeling, have been implicated in mediating this association [121]. Among these, connexin dysregulation, oxidative stress, and glycemic variability-induced cellular injury have emerged as key contributors to the proarrhythmic substrate observed in DM [121].

Both AF and DM are characterized by heightened systemic and tissue-level inflammation, with growing evidence implicating the NLRP3 inflammasome as a central mediator in this pathological interplay [12]. In a pivotal study, Wu et al. [122] investigated the functional significance of the NLRP3–CASP1–galectin-3 axis in diabetic AF using a rabbit model and assessed the therapeutic potential of glibenclamide—an antidiabetic agent with known NLRP3-inhibitory properties. Their findings revealed that glibenclamide treatment effectively mitigated atrial fibrosis, preserved epicardial conduction continuity, restored conduction homogeneity, and reduced AF inducibility in diabetic animals [122]. These beneficial effects were paralleled by the suppression of inflammasome activity, as evidenced by reductions in serum IL-1β and IL-18 levels and the attenuation of atrial CASP1 activity [122]. Furthermore, glibenclamide treatment led to the downregulation of key genes involved in fibrotic and inflammatory signaling—including NLRP3, galectin-3, TGFB1, and CACNA1C (encoding the L-type Ca^2+^ channel)—all of which were markedly upregulated in atrial tissues of diabetic controls [122].

In summary, these findings demonstrate that diabetes promotes activation of the NLRP3 inflammasome–CASP1–galectin-3 axis within atrial tissue, contributing to the initiation of atrial fibrillation. Although atrial remodeling has been more extensively characterized, growing evidence suggests that similar NLRP3-driven electrophysiological alterations also occur in the ventricle, as demonstrated in both ischemic and diabetic cardiomyopathy models [123,124]. Future studies should investigate whether glibenclamide use in diabetic patients correlates with reduced AF incidence, given its inflammasome-inhibitory properties [125]. Notably, persistent atrial injury or worsening glycemic control may diminish glibenclamide efficacy and facilitate the reactivation of inflammasome signaling.

### 3.7. Hypertension and Inflammasome Activation in Atrial Fibrillation

Although the mechanistic link between hypertension and AF remains incompletely defined [126,127,128,129], chronic elevations in arterial pressure are known to increase left atrial wall stress, precipitating structural remodeling marked by chamber dilation and altered atrial compliance [130]. Concurrently, hypertension activates the renin–angiotensin–aldosterone system (RAAS), which contributes to fibrotic and electrical remodeling through maladaptive signaling cascades in the atrial myocardium [130]. A key effector of this process is angiotensin II, which promotes the generation of reactive oxygen species (ROS) via NADPH oxidase 2 activation, facilitating proarrhythmic remodeling and AF onset [131].

In addition to RAAS-mediated fibrosis and conduction impairment, inflammation is increasingly recognized as a pivotal contributor to hypertensive AF [132]. Pressure overload—a hallmark of hypertensive heart disease—has been implicated in the activation of NLRP3 inflammasome signaling within the myocardium [133]. Matsushita et al. [134] investigated this relationship using a transverse aortic constriction (TAC) model of pressure overload in mice deficient in either IL-1β or ASC, a key adaptor protein of the inflammasome complex. In both knockout models, TAC induced significant upregulation of COL1A1 and CTGF, genes associated with extracellular matrix remodeling and fibrosis [134]. Interestingly, while MCP-1 was upregulated in ASC^−/−^ mice, this effect was not observed in IL-1β^−/−^ counterparts, suggesting that some aspects of the fibrotic response may proceed independently of IL-1β [134].

These data indicate that pressure overload-induced atrial remodeling and AF can occur through both NLRP3 inflammasome-dependent and independent pathways. Specifically, IL-1β appears to contribute to atrial fibrogenesis beyond the canonical inflammasome axis, highlighting the complexity of inflammatory signaling in hypertensive AF and the need for nuanced therapeutic approaches targeting both upstream and downstream inflammatory mediators.

### 3.8. Inflammasome-Independent Innate Immune Pathways in Atrial Fibrillation

While the NLRP3 inflammasome has emerged as a central orchestrator of inflammation-induced cardiac remodeling, it is increasingly recognized that inflammasome-independent pathways also contribute to arrhythmogenic processes. Toll-like receptors (TLRs), particularly TLR4, can initiate the NF-κB-dependent transcription of proinflammatory cytokines and fibrotic mediators independent of inflammasome assembly, thereby facilitating structural and electrophysiological remodeling of the atrial and ventricular myocardium [135,136]. Similarly, the cyclic GMP-AMP synthase (cGAS) stimulator of interferon genes (STING) pathway, a cytosolic DNA-sensing mechanism, has been implicated in cardiac inflammation and fibrosis, particularly in the context of mitochondrial DNA release and oxidative stress [137]. The activation of STING can potentiate type I interferon responses and exacerbate cardiomyocyte injury, contributing to adverse remodeling and conduction abnormalities [138,139,140]. Although these pathways may function independently of inflammasomes, emerging evidence suggests considerable crosstalk, particularly through convergent downstream effectors such as NF-κB, IL-6, and ROS [139]. Further elucidation of these parallel and intersecting immune signaling cascades may reveal novel therapeutic opportunities beyond canonical inflammasome inhibition.

## 4. Inflammasome in Ventricular Arrhythmias

Although the majority of inflammasome research in cardiac electrophysiology has focused on atrial fibrillation, a growing body of evidence implicates NLRP3 inflammasome activation in the initiation and perpetuation of ventricular arrhythmias. These arrhythmias often occur in the setting of structural heart disease, ischemia–reperfusion injury, and heart failure—conditions in which inflammation, particularly inflammasome-mediated signaling, is increasingly recognized as a central pathophysiological driver [27,141,142].

### 4.1. Mechanistic Pathways Linking NLRP3 Activation to Ventricular Electrophysiology

NLRP3 activation in ventricular cardiomyocytes results in canonical inflammasome assembly, involving ASC oligomerization and caspase-1 activation, followed by the cleavage and release of IL-1β and IL-18 [143,144]. These cytokines exert profound effects on cardiomyocyte electrophysiology through both direct ionic mechanisms and secondary paracrine signaling:

- **Calcium Dysregulation**: IL-1β has been shown to enhance SR Ca^2+^ leak via RyR2 phosphorylation, contributing to delayed afterdepolarizations (DADs) and triggered activity. This is particularly relevant in the failing myocardium, where calcium mishandling already predisposes to arrhythmogenesis [145].
- **Modulation of Ion Channels**: Inflammasome-derived cytokines suppress repolarizing K^+^ currents (e.g., I_Kr, I_to) and enhance the late sodium current (I_NaL), prolonging action potential duration (APD) and promoting early afterdepolarizations (EADs) [146], a key substrate for torsade de pointes and ventricular tachyarrhythmias [147].
- **Gap Junction Remodeling**: NLRP3-driven inflammation has also been linked to the downregulation of connexin-43 and impaired intercellular coupling [148], which contributes to conduction slowing and reentry formation.

### 4.2. Experimental Evidence

In murine models of pressure overload, ischemia–reperfusion injury, and heart failure, NLRP3 upregulation in the ventricular myocardium correlates with increased arrhythmic burden [149,150,151] (Table 2). The genetic ablation of Nlrp3 or pharmacological inhibition with MCC950 consistently attenuates ventricular arrhythmias, reduces myocardial fibrosis, and preserves conduction velocity [146,152,153]. For instance, in models of diabetic cardiomyopathy, NLRP3 activation was associated with both QT prolongation and the heightened inducibility of ventricular tachycardia, reversible by inflammasome inhibition [154]. Monnerat et al. [154] provided compelling evidence that NLRP3 inflammasome activation within cardiac macrophages promotes ventricular arrhythmogenesis through an IL-1β-dependent mechanism. Specifically, elevated IL-1β production was shown to induce Ca^2+^/calmodulin-dependent protein kinase II (CaMKII) oxidation and phosphorylation in cardiomyocytes, enhancing calcium spark frequency and inhibiting transient outward potassium current (I_to). These changes culminated in prolonged action potential duration and increased susceptibility to ventricular arrhythmias [154]. Notably, the pharmacological inhibition of NLRP3 with MCC950, the blockade of IL-1 signaling with the IL-1 receptor antagonist anakinra, and the genetic silencing of Nlrp3, Casp1, or Il1r1 all attenuated these electrophysiological abnormalities, reinforcing the centrality of the NLRP3–IL-1β axis in arrhythmia pathogenesis [154]. These studies collectively support a causative, rather than merely associative, role for NLRP3 in VA pathophysiology.

However, while these models offer mechanistic clarity, translation into human pathophysiology remains limited. Clinical studies have yet to robustly quantify NLRP3 activation in the ventricular tissue of patients with VAs or sudden cardiac death, and no prospective data exist regarding the antiarrhythmic efficacy of NLRP3 inhibitors in this context. Moreover, the relative contribution of cardiomyocyte versus non-cardiomyocyte (e.g., fibroblast, macrophage) NLRP3 signaling to the arrhythmogenic substrate remains unresolved.

### 4.3. Structural and Metabolic Context

NLRP3 signaling does not act in isolation but intersects with multiple arrhythmogenic pathways [12,155]. In pressure overload and ischemic cardiomyopathy, fibrosis mediated by IL-1β and TGF-β signaling serves as both an anatomic and electrophysiological substrate for reentry [156,157]. In metabolic diseases such as obesity and diabetes, systemic priming of the inflammasome is accompanied by myocardial oxidative stress and mitochondrial dysfunction—key triggers for NLRP3 activation and arrhythmic vulnerability [158].

Importantly, a bidirectional relationship exists between electrical instability and inflammation: while NLRP3 activation promotes electrophysiological dysfunction, sustained arrhythmias can also upregulate inflammasome components via mechanical stretch and ROS production [149,159]. This feedback loop may be particularly relevant in heart failure, where recurrent ventricular arrhythmias exacerbate myocardial injury and systemic inflammation [160].

## 5. Therapeutic Targeting of the NLRP3 Inflammasome

Given its central role in the pathogenesis of atrial fibrillation, the NLRP3 inflammasome represents an attractive therapeutic target. While no NLRP3-specific inhibitors have yet received clinical approval, preclinical studies demonstrate promising efficacy across various inflammatory conditions [161]. Here, we briefly review current strategies and investigational agents aimed at modulating NLRP3 inflammasome signaling.

### 5.1. Targeting Transcriptional and Post-Translational Regulation of NLRP3

One approach to attenuating NLRP3 inflammasome activation involves inhibiting its transcriptional priming, particularly through the suppression of toll-like receptor (TLR)-mediated upregulation. This strategy, while mechanistically logical, poses a risk of off-target effects due to its limited specificity. Inhibitors of interleukin-1 receptor–associated kinase 4 (IRAK4)—a pivotal mediator of TLR-driven NF-κB signaling—are currently in development and may indirectly attenuate NLRP3 activity [162]. However, these agents are not NLRP3-selective and may broadly suppress immune responses, raising concerns about therapeutic precision.

Targeting the post-translational regulation of NLRP3 offers an alternative avenue to suppress inflammasome activation. NLRP3 undergoes multiple phosphorylation events across its leucine-rich repeat (LRR), pyrin domain (PYD), and PYD–NACHT linker regions. Phosphorylation at tyrosine 861 (Y861) and serine 3 (S3) has been shown to inhibit NLRP3 activation, whereas JNK1-dependent phosphorylation at serine 198 (S198) promotes inflammasome assembly and activation [163,164,165]. However, the kinase responsible for S3 phosphorylation remains unidentified, and the therapeutic inhibition of JNK1 may provoke unintended effects due to its broad involvement in cellular signaling networks [165].

In parallel, NLRP3 activity is regulated by ubiquitination. Enhanced ubiquitination suppresses NLRP3 activation, while deubiquitylation facilitates its stabilization and inflammasome assembly. Accordingly, strategies that promote NLRP3 degradation or inhibit its deubiquitinases may offer therapeutic potential. Several deubiquitinase inhibitors are currently in development [166], though their applicability to atrial fibrillation and their selectivity profiles require further investigation. As with other upstream approaches, achieving target specificity remains a fundamental challenge.

Inhibiting downstream effectors of the NLRP3 inflammasome, such as IL-1β, offers another therapeutic approach for inflammatory conditions like AF [167]. However, this strategy faces significant limitations due to poor specificity. The broad inhibition of inflammasome components may suppress all inflammasome activity, compromising host immunity and increasing infection risk [167]. Moreover, IL-1β is produced by multiple inflammasome pathways, which could diminish the selectivity and therapeutic efficacy of NLRP3-specific interventions [167].

### 5.2. Targeting IL-1β as a Downstream Effector of NLRP3 Activation

IL-1β, a key proinflammatory cytokine processed through NLRP3 inflammasome activation, represents a logical therapeutic target in mitigating NLRP3-driven pathology [168]. However, the inhibition of IL-1β signaling—typically via IL-1 receptor (IL-1R) blockade—lacks pathway specificity, as it disrupts all IL-1R-mediated responses regardless of inflammasome origin. Several agents are currently approved for clinical use: anakinra, a recombinant IL-1R antagonist [169]; canakinumab, a monoclonal antibody that selectively neutralizes IL-1β; and rilonacept, a fusion protein that sequesters both IL-1α and IL-1β [170]. While anakinra is approved for NLRP3-related diseases such as rheumatoid arthritis, its potential in atrial fibrillation remains untested [171]. Moreover, its short plasma half-life necessitates frequent dosing, which may limit its clinical utility in chronic conditions [172].

Canakinumab, a long-acting monoclonal antibody with a half-life of approximately 26 days, has been approved for the treatment of caspase-driven inflammatory conditions such as atherosclerosis, gout, and arthritis [173,174]. Despite its clinical utility, its use is limited by adverse effects, including injection site reactions and discomfort upon administration [175,176]. These limitations underscore the need for novel, orally bioavailable small-molecule inhibitors that selectively target NLRP3 inflammasome signaling in atrial fibrillation, offering improved tolerability and therapeutic precision.

### 5.3. Targeting ASC and Caspase-1 in Inflammasome Signaling

As a central adaptor within the NLRP3 inflammasome complex, ASC facilitates the recruitment and activation of caspase-1. Inhibiting ASC may theoretically disrupt inflammasome assembly and downstream inflammatory signaling [168]. However, ASC is a shared component across multiple inflammasome pathways, raising concerns about target specificity. Moreover, limited mechanistic insight into ASC oligomerization and regulation has hindered the development of selective inhibitors, and no pharmacologic ASC-targeting agents have reached clinical application to date. Caspase-1 inhibition has been explored using peptidomimetic small molecules such as VX-765 and its precursor VX-740 (pralnacasan), both of which are prodrugs metabolized to active compounds (VRT-043198 and VRT-18858, respectively) via plasma esterases [177,178]. These agents have shown promise in early-phase clinical trials for inflammatory disorders including psoriasis and epilepsy. However, development was discontinued due to hepatotoxicity concerns [179]. Additionally, given caspase-1’s broader role in multiple inflammasome pathways, its inhibition could compromise host immune defense and increase susceptibility to infections. Altogether, while ASC and caspase-1 represent mechanistically rational targets within the inflammasome cascade, their therapeutic exploitation in atrial fibrillation remains limited. Future efforts should focus on developing selective, safe inhibitors with relevance to cardiac-specific inflammatory signaling.

### 5.4. Colchicine as a Modulator of Inflammasome-Driven Inflammation and Fibrosis

Colchicine, a plant-derived alkaloid traditionally used for its anti-inflammatory properties, has garnered interest as a potential therapeutic agent in the prevention and treatment of early and postoperative AF [180,181]. Clinical observations have highlighted its efficacy in modulating inflammatory responses associated with AF onset. In a preclinical model of sterile pericarditis, Wu et al. [182] demonstrated that colchicine, administered at an optimal dose of 0.5 mg/kg, significantly reduced AF inducibility and duration. Mechanistically, colchicine attenuated neutrophil infiltration and downregulated the expression of proinflammatory and profibrotic mediators in atrial tissue, including TNF, TGFB1, IL6, STAT3, and extracellular matrix-related genes [182]. Importantly, colchicine inhibited the IL-1β-driven upregulation of IL6 and suppressed NF-κB phosphorylation and activation, implicating a blockade of the IL-1β–IL-6 inflammatory axis [182]. These effects collectively contributed to the attenuation of atrial fibrosis and electrical remodeling. These findings support the therapeutic potential of colchicine in targeting upstream NLRP3 inflammasome activation and downstream IL-1β signaling, offering a mechanistically grounded approach to mitigate inflammation-induced atrial remodeling and AF vulnerability.

### 5.5. Salvianolate as a Modulator of NLRP3-Driven Atrial Remodeling

Salvianolate, a bioactive compound extracted from Salvia miltiorrhiza Bunge, is clinically employed in China for the treatment of cardiovascular diseases [183]. In a murine model of post-myocardial infarction atrial fibrillation, Qiu et al. [184] demonstrated that salvianolate significantly improved cardiac function and reduced AF burden. Notably, it shortened AF duration and P-wave length and mitigated atrial dilation, hypertrophy, and interstitial fibrosis [184]. Mechanistically, these therapeutic effects were linked to the dual inhibition of key profibrotic and proinflammatory pathways: suppression of the TGF-β/Smad2/3 signaling axis, which drives collagen deposition, and downregulation of the TXNIP–NLRP3–IL-1β/IL-18 cascade, a critical inflammatory route implicated in atrial remodeling [184].

### 5.6. Sodium–Glucose Cotransporter 2 Inhibitors

Sodium–glucose cotransporter 2 (SGLT2) inhibitors, originally developed as glucose-lowering agents, have emerged as multifaceted cardioprotective drugs with pleiotropic effects extending beyond glycemic control [185,186,187,188,189,190,191]. Mounting evidence suggests that SGLT2 inhibitors exert robust anti-inflammatory, antifibrotic, and antioxidative actions, many of which converge on suppression of the NLRP3 inflammasome—a central node in AF pathophysiology [192,193,194,195]. Consistently, a recent meta-analysis of 38 randomized controlled trials involving over 88,000 patients demonstrated that SGLT2 inhibitor therapy significantly reduced the risk of atrial arrhythmias (OR: 0.85, 95% CI: 0.75–0.98) and sudden cardiac death (OR: 0.72, 95% CI: 0.55–0.94), further substantiating their emerging role in rhythm modulation and inflammasome-related substrate modification [196].

Mechanistically, SGLT2 inhibitors attenuate intracellular sodium and calcium overload by reducing cytosolic Na^+^ through inhibition of the sodium–hydrogen exchanger (NHE1) [197,198]. This ionic modulation indirectly stabilizes mitochondrial function, mitigating ROS generation—a key trigger for NLRP3 activation. In both diabetic and non-diabetic preclinical models, treatment with empagliflozin or dapagliflozin resulted in the downregulation of NLRP3, caspase-1, and IL-1β in cardiac tissues, suggesting a conserved anti-inflammasome effect across metabolic backgrounds [199,200,201]. Emerging evidence also suggests that SGLT2 inhibitors may indirectly modulate epigenetic programs relevant to atrial remodeling, including microRNA expression and chromatin accessibility, further linking metabolic therapy to substrate modification in AF [20].

In the setting of AF, these agents may offer dual benefit: (1) electrophysiologic stabilization through the amelioration of Ca^2+^ mishandling and oxidative stress, which disrupts arrhythmogenic calcium leak from the sarcoplasmic reticulum and (2) structural remodeling attenuation by blunting the inflammasome-induced activation of profibrotic pathways (e.g., TGF-β/Smad and galectin-3 signaling) [202,203]. Importantly, SGLT2 inhibition has been shown to decrease circulating levels of proinflammatory cytokines such as IL-6 and TNF-α and reduce the atrial expression of fibrotic genes (e.g., COL1A1, ACTA2) [204,205], providing further evidence for their upstream modulation of inflammasome-mediated remodeling [206]. Emerging data also suggest that SGLT2 inhibitors may limit epicardial adipose tissue inflammation and its paracrine impact on atrial myocardium—an increasingly recognized contributor to AF substrate formation [203,207,208].

Given the shared pathophysiology between AF and HFpEF, SGLT2 inhibitors may reduce AF susceptibility not only through anti-inflammatory and metabolic effects but also by lowering left atrial pressure and wall stress via improved diastolic function and volume unloading [209,210]. This hemodynamic benefit likely contributes to their emerging antiarrhythmic profile in patients with elevated filling pressures. Although mechanistic insights continue to evolve, the integration of SGLT2 inhibitors into AF management holds significant promise, particularly in patients with diabetes, heart failure, or obesity—conditions strongly associated with heightened NLRP3 activity. Future studies should evaluate whether their anti-inflammasome effects translate into meaningful reductions in AF incidence, burden, or recurrence after ablation.

### 5.7. Mitochondrial Antioxidants: Targeted Redox Modulation to Disrupt AF-Linked Inflammation

In parallel with SGLT2 inhibitors, mitochondrial-targeted antioxidants such as MitoTEMPO and MitoQ effectively prevent AF in preclinical models by quenching mitochondrial ROS—the primary upstream trigger of NLRP3 inflammasome activation. In canine and murine AF models, MitoTEMPO reduced spontaneous and sustained AF episodes, while MitoQ preserved mitochondrial structure, attenuated action potential shortening, normalized L-type Ca^2+^ currents, and limited atrial fibrosis; notably, these benefits were achieved without systemic immunosuppression [211]. This targeted redox modulation highlights mitochondrial antioxidants as a mechanistically precise and immunologically safer therapeutic complement to broader anti-inflammatory approaches in AF.

### 5.8. Pro-Resolving Inflammatory Pathways

Emerging evidence suggests that activating endogenous pro-resolving pathways—via resident CCR2^−^ cardiac macrophages or specialized lipid mediators such as resolvins—can mitigate AF-associated inflammation with minimal systemic side effects. CCR2^−^ resident macrophages are critical for inflammation resolution, facilitating efferocytosis and secreting anti-inflammatory cytokines like IL-10 and lipoxins to promote tissue repair and maintain electrophysiological homeostasis [212]. In parallel, resolvin-D1 treatment has been shown in rodent post-myocardial infarction and right-heart disease models to reduce atrial fibrosis, shift macrophage populations from proinflammatory (M1) to anti-inflammatory (M2), improve conduction, and suppress AF vulnerability [124]. Thus, engaging these pro-resolving cascades offers a promising, homeostatic strategy to counteract arrhythmogenic remodeling without the risks associated with broad immunosuppression.

## 6. Future Directions and Conclusions

Future research must focus on unraveling the cell-type-specific roles of NLRP3 inflammasome activation within the atrial and ventricular myocardium. Dissecting the relative contributions of cardiomyocytes, fibroblasts, macrophages, and epicardial adipocytes will be essential for the development of precision-targeted therapies. Equally important is the advancement of non-invasive biomarkers that reflect inflammasome activation in vivo—tools that could inform risk stratification, monitor disease progression, and guide anti-inflammatory interventions. The pharmacologic targeting of inflammasome signaling remains a promising yet underdeveloped area in arrhythmia therapeutics. While existing commercially available agents offer preliminary evidence of efficacy, their broad anti-inflammatory profiles may limit specificity. In contrast, SGLT2 inhibitors—now widely adopted in cardiovascular medicine—demonstrate favorable effects on NLRP3 suppression, atrial structural remodeling, and arrhythmia prevention, particularly in patients with diabetes or heart failure. Ongoing and future randomized trials should evaluate their impact on atrial fibrillation burden, recurrence post-ablation, and progression from paroxysmal to persistent forms. In parallel, deeper exploration of the epigenetic mechanisms governing inflammasome activation—such as the IL-6–STAT3–miR-21 axis—may reveal novel modulators of arrhythmogenic inflammation and open avenues for transcriptional and post-transcriptional therapeutic interventions.

In conclusion, the NLRP3 inflammasome sits at the intersection of inflammation, fibrosis, and electrophysiological remodeling, forming a unifying mechanistic link in the pathogenesis of atrial and ventricular arrhythmias. While much remains to be clarified, the growing body of experimental and translational evidence strongly supports the inflammasome as a viable therapeutic target. Integrating immunomodulation into the arrhythmia treatment paradigm may not only reduce arrhythmic risk but also attenuate the broader trajectory of structural heart disease. Moving forward, the integration of molecular insights into clinical practice will be pivotal in redefining treatment strategies within the framework of precision arrhythmia management.

## Figures and Tables

**Table 1:** Key preclinical and translational studies elucidating the role of inflammasome activation in atrial fibrillation pathophysiology.

Author, Year	Study Model	Cell Type(s) Involved	Key Findings	Implications for AF
Yao et al., 2018 [19]	Human, Dog, and Mouse models (including CM-specific knock-in and CREM-TG mice)	Cardiomyocytes	Enhanced NLRP3 inflammasome activation in atrial cardiomyocytes from AF patients and animal models; CM-specific NLRP3 activation induced ectopic activity, shortened AERP, structural remodeling, and increased AF susceptibility. Inhibition (MCC950, AAV-shRNA, or knockout) reduced AF burden.	NLRP3 inflammasome in cardiomyocytes promotes both arrhythmic triggers and substrate for AF; targeting NLRP3 may represent a novel therapeutic approach.
Heijman et al., 2020 [80]	Human atrial tissue from cardiac surgery patients	Cardiomyocytes	Patients developing POAF showed increased atrial NLRP3-inflammasome activation and CaMKII-mediated RyR2 dysfunction; Ca^2+^-handling abnormalities, including SR Ca^2+^-leak and spontaneous Ca^2+^-release events, were present preoperatively. Acute IL-1β exposure exacerbated arrhythmogenic Ca^2+^ disturbances in atrial myocytes.	NLRP3/CaMKII signaling constitutes a latent arrhythmogenic substrate for POAF, unmasked by postoperative inflammation; targeting these pathways may prevent POAF and long-term AF progression.
Fender et al., 2020 [76]	Mouse (HFD model), Human atrial tissue, Human ventricular fibroblasts	Cardiac fibroblasts, cardiomyocytes	PAR4 expression is upregulated in diabetic hearts and mediates thrombin-induced activation of canonical NLRP3 inflammasome via caspase-1, IL-1β, and GSDMD in both mouse and human cardiac tissues. Genetic deletion or pharmacologic inhibition of PAR4 blunted this pathway.	Diabetes-associated upregulation of PAR4 links hypercoagulability to sterile cardiac inflammation through the NLRP3 inflammasome; PAR4 antagonists may mitigate thromboinflammatory contributions to AF substrate development.
Song et al., 2024 [81]	Mouse (WT and Aim2^−/−^), atrial myocytes	Atrial cardiomyocytes	High-protein diet (HPD) enhanced AF inducibility via AIM2 inflammasome activation; AIM2^−/−^ mice were protected from HPD-induced AF. HPD promoted mitochondrial ROS, cytoplasmic dsDNA, and abnormal SR Ca^2+^ release, which were suppressed in AIM2-deficient mice.	AIM2 inflammasome links dietary triggers (HPD) to arrhythmogenesis via mitochondrial stress and Ca^2+^ dysregulation; AIM2 may serve as a novel therapeutic target for metabolically driven AF.
Yao et al., 2016 [87]	Mouse (CM-specific NLRP3 A350V knock-in, CREM-IbΔC-X Tg)	Cardiomyocytes, Fibroblasts, Macrophages	CM-specific activation of NLRP3 inflammasome increased AF inducibility and premature atrial contractions. Upregulation of active caspase-1, IL-1β, and collagen 1a indicated fibroblast and macrophage activation. NLRP3 deletion in CREM-Tg mice reduced spontaneous AF.	Constitutive NLRP3 activation in cardiomyocytes drives AF initiation and promotes atrial remodeling; therapeutic targeting of NLRP3 could interrupt inflammatory and fibrotic AF substrates.
Huang et al., 2016 [90]	Rat sterile pericarditis model, primary cultured cardiac fibroblasts	Cardiac fibroblasts	STAT3 and miR-21 form a positive feedback loop that promotes atrial fibrosis. Inhibition of STAT3 (S3I-201) or miR-21 (antagomir-21) reduced atrial fibrosis, conduction inhomogeneity, and inducible AF. IL-6 stimulated CF activation via increased STAT3 phosphorylation and miR-21 expression; blockade of either reduced fibrotic gene expression and fibroblast proliferation.	While not directly implicating inflammasomes, this study highlights IL-6/STAT3/miR-21 signaling as a crucial inflammatory–fibrotic axis in AF substrate formation; targeting this pathway could indirectly modulate inflammasome-mediated profibrotic signaling.
Li et al., 2023 [102]	Human, Canine, and FB-specific NLRP3 KI Mouse Model (Tcf21iCre:Nlrp3A350V)	Cardiac Fibroblasts	NLRP3 and IL1B upregulated in human atrial FBs from AF patients and canine AF model. FB-specific NLRP3 activation in mice caused atrial dilation, fibrosis, hypocontractility, and enhanced AF inducibility. Connexin 43 remodeling and impaired intercellular communication contributed to reduced conduction velocity.	Fibroblast-restricted NLRP3 activation drives atrial cardiomyopathy and arrhythmogenesis via fibrotic and gap junction remodeling; suggests NLRP3 as a unifying target across cardiac cell types in AF therapy.
Scott et al., 2021 [103]	Human, Sheep, Mouse (WT and NLRP3^−/−^ with HFD)	Cardiomyocytes	Obesity enhanced atrial NLRP3 inflammasome activation in humans, sheep, and HFD-fed mice; NLRP3^−/−^ mice were protected from AF inducibility, atrial refractoriness shortening, abnormal Ca^2+^ handling, and atrial fibrosis. ER stress and Kv1.5 upregulation contributed to the proarrhythmic substrate.	NLRP3 inflammasome mediates obesity-induced atrial arrhythmogenesis through inflammatory, electrical, and structural remodeling; targeting NLRP3 may mitigate AF risk in obese individuals.

Abbreviations: AF, atrial fibrillation; AIM2, absent in melanoma 2; AERP, atrial effective refractory period; CaMKII, calcium/calmodulin-dependent protein kinase II; CM, cardiomyocyte; CREM-TG, cAMP response element modulator transgenic; dsDNA, double-stranded DNA; FB, fibroblast; HFD, high-fat diet; HPD, high-protein diet; IL, interleukin; KI, knock-in; KO, knockout; miR, microRNA; NLRP3, NOD-like receptor family pyrin domain containing 3; PAR4, protease-activated receptor 4; POAF, postoperative atrial fibrillation; ROS, reactive oxygen species; SR, sarcoplasmic reticulum; STAT3, signal transducer and activator of transcription 3; and WT, wild type.

**Table 2:** Key experimental studies investigating inflammasome activation in ventricular arrhythmogenesis.

Author, Year	Study Model	Cell Type(s) Involved	Key Findings	Implications for Ventricular Arrhythmias
Suetomi et al., 2018 [133]	Mouse (CM-specific CaMKIIδ KO, TAC model), Human	Cardiomyocytes	Pressure overload triggered NLRP3 inflammasome activation in cardiomyocytes via CaMKIIδ-mediated NFκB and ROS signaling. This led to early cytokine production, macrophage recruitment, fibrosis, and ventricular dysfunction. CaMKIIδ deletion or NLRP3 inhibition prevented remodeling.	CM-specific NLRP3 activation initiates inflammatory cascades that promote adverse ventricular remodeling and dysfunction; early inhibition may prevent heart failure and reduce arrhythmogenic substrate development.
Jiang et al., 2022 [146]	Mouse (TAC-induced HF model)	Cardiomyocytes	MCC950, a selective NLRP3 inhibitor, reduced QTc and APD90, suppressed VA inducibility, and ameliorated HF-induced cardiac hypertrophy, fibrosis, and ion channel remodeling (Kv4.2, KChIP2, Cav1.2). MCC950 downregulated NLRP3, ASC, caspase-1, IL-1β, and IL-18 expression, indicating suppression of inflammasome signaling.	NLRP3 inflammasome inhibition by MCC950 prevents electrical and structural remodeling and reduces susceptibility to HF-induced ventricular arrhythmias; suggests translational potential for targeted anti-inflammatory therapy in arrhythmia prevention.
Higashikuni et al., 2023 [149]	Mouse (WT, Nlrp3^−/−^, P2rx7^−/−^, Slc17a9 conditional KO), Human heart tissue, in vitro cardiomyocyte, fibroblast, endothelial models	Cardiomyocytes, Fibroblasts, Endothelial Cells	Pressure overload induces cardiac NLRP3 activation through ATP release from sympathetic efferent nerves via P2X7 receptors. NLRP3 deficiency or ATP/P2X7 blockade reduced IL-1β, hypertrophy, fibrosis, macrophage infiltration, and capillary density. Neural signals (afferent/efferent) and β-blockers modulate inflammasome activity.	Reveals a novel heart–brain axis in inflammasome-driven cardiac remodeling; modulation of neural pathways and NLRP3 inhibition may prevent structural arrhythmogenic substrate formation.
Toldo et al., 2016 [150]	Mouse (Iischemia–reperfusion model, ICR male mice)	Cardiomyocytes	NLRP3 expression and caspase-1 activity increased progressively after reperfusion. Pharmacologic NLRP3 inhibition reduced infarct size and caspase-1 activation when administered at or 1 h after reperfusion. No benefit observed if administered 3 h post-reperfusion. Infarct size reduction confirmed even in prolonged ischemia model.	NLRP3 activation exacerbates post-reperfusion myocardial injury and inflammation. Timely NLRP3 inhibition may preserve ventricular integrity and limit arrhythmogenic remodeling following AMI.
Gao et al., 2019 [152]	Mouse (MI model via coronary ligation), in vitro cardiac fibroblast model	Cardiomyocytes, Cardiac Fibroblasts	MCC950 significantly reduced myocardial fibrosis, preserved ejection fraction, and suppressed expression of NLRP3, IL-1β, and IL-18. In vitro, MCC950 attenuated hypoxia-induced fibroblast activation and inflammatory cytokine production without affecting proliferation.	Post-MI NLRP3 activation in fibroblasts and cardiomyocytes contributes to inflammation and remodeling. MCC950 reduces fibrosis and may help prevent arrhythmogenic substrate development post-infarction.
Monnerat et al., 2016 [154]	Mouse (streptozotocin-induced DM model), Rat and Human cardiomyocytes	Macrophages, Cardiomyocytes	TLR2/NLRP3 activation in diabetic heart macrophages promotes IL-1β production, which prolongs QT/APD, reduces Ito, increases Ca^2+^ sparks, and enhances spontaneous arrhythmias. IL-1β-induced CaMKII oxidation/phosphorylation underlies arrhythmogenic remodeling. Genetic or pharmacologic targeting (IL-1R KO, NLRP3 KO, Casp1 KO, Anakinra, MCC950) prevented arrhythmias.	Demonstrates inflammasome-mediated macrophage–cardiomyocyte crosstalk in diabetic arrhythmogenesis; highlights therapeutic potential of IL-1 axis and NLRP3 inhibition in preventing ventricular arrhythmias in diabetes.

Abbreviations: APD, action potential duration; APD90, action potential duration at 90% repolarization; CaMKII, calcium/calmodulin-dependent protein kinase II; Casp1, caspase-1; CM, cardiomyocyte; DM, diabetes mellitus; HF, heart failure; IL, interleukin; IL-1Ra, interleukin-1 receptor antagonist (anakinra); Ito, transient outward potassium current; KO, knockout; MI, myocardial infarction; MCC950 (CRID3), selective NLRP3 inflammasome inhibitor; NLRP3, NOD-like receptor pyrin domain-containing protein 3; QTc, corrected QT interval; TAC, transverse aortic constriction; TLR, toll-like receptor; and WT, wild type.
